# Glycosylphosphatidylinositol-Anchored Immunoglobulin Superfamily Cell Adhesion Molecules and Their Role in Neuronal Development and Synapse Regulation

**DOI:** 10.3389/fnmol.2017.00378

**Published:** 2017-11-15

**Authors:** Rui P. A. Tan, Iryna Leshchyns’ka, Vladimir Sytnyk

**Affiliations:** School of Biotechnology and Biomolecular Sciences, The University of New South Wales, Sydney, NSW, Australia

**Keywords:** cell adhesion molecules, neuronal, GPI anchor, synapses, neurite outgrowth, synaptic plasticity (LTP/LTD), learning and memory

## Abstract

Immunoglobulin superfamily (IgSF) cell adhesion molecules (CAMs) are cell surface glycoproteins that not only mediate interactions between neurons but also between neurons and other cells in the nervous system. While typical IgSF CAMs are transmembrane molecules, this superfamily also includes CAMs, which do not possess transmembrane and intracellular domains and are instead attached to the plasma membrane via a glycosylphosphatidylinositol (GPI) anchor. In this review, we focus on the role GPI-anchored IgSF CAMs have as signal transducers and ligands in neurons, and discuss their functions in regulation of neuronal development, synapse formation, synaptic plasticity, learning, and behavior. We also review the links between GPI-anchored IgSF CAMs and brain disorders.

## Introduction

Cell adhesion molecules (CAMs) are expressed across all cell types. In the nervous system, multiple families of CAMs are expressed in neurons, including integrins, cadherins, selectins, neuroligins, neurexins, and the immunoglobulin superfamily (IgSF) of CAMs (Brümmendorf and Rathjen, 1993; Chothia and Jones, 1997; Buckley et al., 1998; Südhof, 2008; Sytnyk et al., 2017). These molecules play numerous roles in the developing and mature nervous system by regulating growth and branching of neurites, navigating growing axons and dendrites to the appropriate targets, regulating formation and maturation of synaptic contacts, and maintaining synapse function and plasticity during learning and memory formation.

**FIGURE 1:**
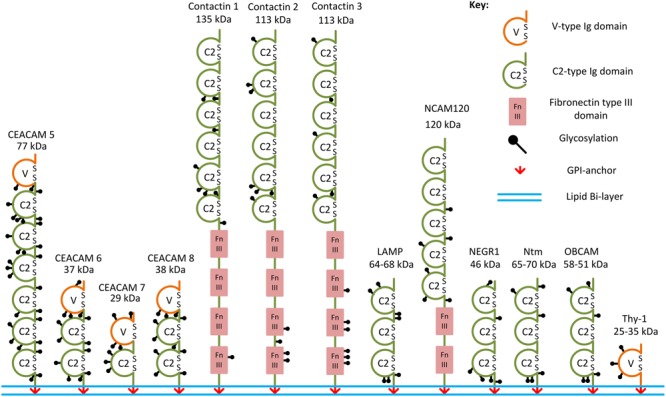
Examples of the GPI-anchored IgSF CAMs. Structural domains and putative glycosylation sites in the GPI-anchored IgSF CAMs are represented according to the Uniprot database. SS, disulfide bridges present in Ig domains.

Typically, and for some families exclusively, CAMs are transmembrane proteins. While the extracellular domains of these molecules mediate interactions not only between neurons but also between neurons and other cells by interacting with the same molecules or other types of molecules either on the membranes of other cells or in the extracellular matrix, the intracellular domains are involved in interactions with the cytoskeleton and signal transduction (Leshchyns’ka and Sytnyk, 2016). However, CAMs can also be anchored to the plasma membranes via a glycosylphosphatidylinositol (GPI) anchor, with the highest number of the GPI-anchored CAMs within the IgSF (**Figure 1** and **Table 1**). Although these proteins do not possess intracellular domains, their functions are not limited to mediating cell adhesion only. In this review, we summarize the role GPI-anchored IgSF CAMs have as signal transducers, ligands, synapse formation regulators, as well as their role in synaptic plasticity and brain disorders.

**Table 1:** List of the GPI-anchored IgSF CAMs and their functions in neurite outgrowth and synapse formation and plasticity.

IgSF member	Number and type of Ig domains	Homophilic interaction	Examples of heterophilic binding partners	Function in neurite outgrowth as a receptor	Function in neurite outgrowth as a ligand	Role in synapse formation	Changes in LTP/LTD in knock-out (KO) or transgenic (TG) mice
**CEACAM5**/ CD66e/CEA	One V-type and six C2-type domains (Oikawa et al., 1987).	In *trans* (Zhou et al., 1993; Taheri et al., 2000).	*Trans* – NA;*Cis* – NA;*ECM* – NA.	NA	NA	NA	NA
**CEACAM6**/ CD66c/NCA	One V-type and two C2-type domains (Oikawa et al., 1987).	NA	*Trans* – CEACAM8 (Oikawa et al., 1991);*Cis* – NA;*ECM* – NA.	NA	NA	NA	NA
**CEACAM7**/ CGM2	One V-type and one C2-type domains (Oikawa et al., 1987).	NA	*Trans* – NA; *Cis* – NA;*ECM* – NA.	NA	NA	NA	NA
**CEACAM8**/ CD66b/CGM6	One V-type and two C2-type domains (Oikawa et al., 1987).	NA	*Trans* – CEACAM6 (Oikawa et al., 1991);*Cis* – NA;*ECM* – NA.	NA	NA	NA	NA
**Contactin1** / F3/F11/ Contactin	Six C2-type domains (Ranscht, 1988; Brümmendorf et al., 1989).	NA	*Trans* – NrCAM, NgCAM (Morales et al., 1993); – PTPRZ (Peles et al., 1995; Bouyain and Watkins, 2010); – CASPR2 (Rubio-Marrero et al., 2016); – Notch (Hu et al., 2003). *Cis* – CASPR (Peles et al., 1997); – L1 (Olive et al., 2002). *ECM* – Chondroitin sulfate E (Mikami et al., 2009); – Tenascin-R (Norenberg et al., 1995).	– Promotes (Mikami et al., 2009); – Promotes filopodia and lamellipodia (Zacharias, 2002).	– Promotes (Treubert and Brümmendorf, 1998).	NA	KO: – LTP in CA1 is normal, – LTD in CA1 is impaired (Murai et al., 2002); TG (overexpressing): – LTP in CA1 is normal at 5 months, increased at 12 months of age (Puzzo et al., 2013).
**Contactin2**/ Tag1/axonin1	Six C2-type domains (but see text) (Zuellig et al., 1992; Freigang et al., 2000).	in *trans* (Rader et al., 1993; Felsenfeld et al., 1994; Malhotra et al., 1998; Lustig et al., 1999; Traka et al., 2003).	*Trans* –NrCAM (Lustig et al., 1999); – NgCAM (Fitzli et al., 2000); – L1 (Kuhn et al., 1991; Stoeckli et al., 1991; Felsenfeld et al., 1994; Wang et al., 2011); – β1 integrin (Felsenfeld et al., 1994); – APP (Ma et al., 2008). *Cis* – L1 (Malhotra et al., 1998); – NgCAM (Buchstaller et al., 1996); – CASPR2 (Traka et al., 2003). *ECM* – NA.	– Promotes (Buchstaller et al., 1996; Lustig et al., 1999).	– Promotes (Kuhn et al., 1991; Stoeckli et al., 1991; Kasahara et al., 2002).	NA	NA
**Contactin3**/ BIG-1	Six C2-type domains (Yoshihara et al., 1994).	NA	*Trans* – PTPRG (Bouyain and Watkins, 2010). *Cis* – PTPRG (Bouyain and Watkins, 2010; Nikolaienko et al., 2016). *ECM* – NA.	NA	– Promotes (Yoshihara et al., 1994).	NA	NA
**Contactin4**/ BIG-2	Six C2-type domains (Yoshihara et al., 1995).	NA	*Trans* – PTPRG (Bouyain and Watkins, 2010). *Cis* – PTPRG (Bouyain and Watkins, 2010). *Soluble* – NA.	NA	– Promotes (Yoshihara et al., 1995)	NA	NA
**Contactin5**/ NB-2	Six C2-type domains (Ogawa et al., 1996)	NA	*Trans* – PTPRG (Bouyain and Watkins, 2010). *Cis* – PTPRG (Bouyain and Watkins, 2010); – APLP1 (Shimoda et al., 2012). *ECM* – NA.	NA	– Promotes (Bouyain and Watkins, 2010; Mercati et al., 2013).	NA	NA
**Contactin6**/ NB-3	Six C2-type domains (Ogawa et al., 1996).	NA	*Trans* – PTPRG (Bouyain and Watkins, 2010). *Cis* – PTPRG (Bouyain and Watkins, 2010); – CHL1 (Ye et al., 2008). *ECM* – NA.	NA	– Promotes (Bouyain and Watkins, 2010; Mercati et al., 2013).	NA	NA
**IgLON5**	NA	NA	*Trans* – NA;*Cis* – NA;*ECM* – NA.	NA	NA	NA	NA
**LAMP**/ LSAMP/IGLON3	Three C2-type domains (Pimenta et al., 1996).	in *trans* (Zhukareva and Levitt, 1995; Lodge et al., 2000; Gil et al., 2002).	*Trans* – Ntm (Gil et al., 2002). *Cis* – OBCAM (Reed et al., 2004). *ECM* – NA.	– Promotes (Eagleson et al., 2003).	– Promotes via the first Ig-like domain (Eagleson et al., 2003; Sanz et al., 2015); – Inhibits via the second Ig-like domain (Eagleson et al., 2003).	– Promotes (Hashimoto et al., 2009).	KO: – LTP in CA1 is decreased (Qiu et al., 2010).
**NEGR1**/ Klion/IGLON4	Three C2-type domains (Funatsu et al., 1999).	in *trans* (Miyata et al., 2003a) But see, (Marg et al., 1999).	*Trans* – Ntm, LAMP (Marg et al., 1999); – FGFR2 (Pischedda and Piccoli, 2015); – ObCAM (Miyata et al., 2003a). *Cis* – NA; *ECM –* NA.	NA	– Promotes (Pischedda and Piccoli, 2015; Sanz et al., 2015).	– Inhibits (Hashimoto et al., 2009).	NA
**Ntm**/ NT/IGLON2	Three C2-type domains (Struyk et al., 1995).	in *trans* (Lodge et al., 2000).	Trans – LAMP (Gil et al., 2002) *Cis* – NA; *ECM –* NA.	– Promotes (Gil et al., 1998, 2002).	– Promotes (Sanz et al., 2015).	NA	NA
**OBCAM**/ OPCML/IGLON1	Three C2-type domains (Hachisuka et al., 1996).	in *trans* (Lodge et al., 2000).	*Trans* – NEGR1 (Miyata et al., 2003a). *Cis* – LAMP (Lodge et al., 2000). *ECM –* NA.	NA	– Promotes (Sanz et al., 2015).	– Promotes (Li et al., 2006; Yamada et al., 2007; Hashimoto et al., 2009).	NA
**Thy1**/ CD90/CDw90	One V-type domain (Williams and Gagnon, 1982).	In *trans* (Mahanthappa and Patterson, 1992).	*Trans* – β3 integrin (Leyton et al., 2001). *Cis* – Integrins (Kuroiwa et al., 2012). *ECM* – NA.	– Promotes when binds to antibodies (Leifer et al., 1984; Messer et al., 1984; Chen et al., 2005); – Inhibits when binds to αVβ3 integrins (Herrera-Molina et al., 2012).	NA	NA	KO: – LTP in CA1 is normal; – LTP in dentate gyrus is impaired (Zacco et al., 1990).

### GPI-Anchored IgSF CAMs and Their Homophilic and Heterophilic Interactions

**FIGURE 2:**
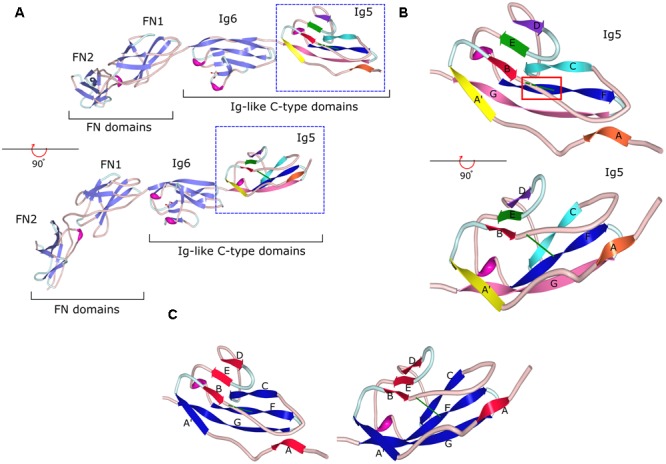
Crystal structure of the fragment of contactin-3 containing Ig-like domains 5 and 6 and fibronectin type III domains 1 and 2. **(A)** fibronectin type III (FN) and immunoglobulin-like (Ig) C2-type domains of contactin-3 are shown as a ribbon diagram in two views flipped 90° to demonstrate the β-sheet orientation and disulfide bonds in Ig domains (green lines). β-Sheet strands are shown as light purple arrows in Ig6, FN2, and FN1 and multicolored arrows in Ig5. Coils are in light pink and turns are in light blue. **(B)** A ribbon diagram of the Ig5 domain with one β-sheet represented by strands A (orange), B (red), E (green), and D (purple) and the second sheet represented by strands A′ (yellow), C (cyan), F (blue), and G (pink). Strands E and F are connected by a short helix (in magenta). Two views flipped 90° are shown to demonstrate the β-sheet orientation and the disulfide bond represented as a green line in both views and highlighted by a red box in the upper view. Dashed green line represents the disulfide bond behind the B–C coil. **(C)** A ribbon diagram of the Ig5 domain with β-sheet strands re-colored to demonstrate the two sheets, one comprised of strands A, B, E, and D (red) and another comprised of strands A′, C, F, and G (blue) with the disulfide bond (green) holding the two sheets together. Image of PDB ID 5I99 (Nikolaienko et al., 2016) created with Protein Workshop (Moreland et al., 2005).

Immunoglobulin superfamily CAMs are identified by the presence of immunoglobulin (Ig)-like domains in their ectodomains. There are several types of Ig domains present in IgSF CAMs including C2-, V-, and I-type (Williams and Barclay, 1988; Harpaz and Chothia, 1994). The V-type is similar to the variable V-domain in Igs, whereas the C2 type is similar to C1-domains in Igs (Barclay, 2003). The I-type (I for intermediate) shares similarities with C1- and V-type domains and was initially identified in Telokin, an intracellular smooth muscle protein (Harpaz and Chothia, 1994). GPI-anchored IgSF CAMs differ in the numbers of V-, C2-, or I-type Ig domains present in their ectodomains. For example, only one V-type Ig domain is present in Thy-1, while there are three C2-type Ig domains present in neuronal growth regulator 1 (NEGR1), and six C2-type domains present in contactin-1, -2, -3 (Ranscht, 1988; Williams and Barclay, 1988; Brümmendorf et al., 1989; Zuellig et al., 1992; **Figure 1**). The first four Ig domains of contactin-2 have also been classified as I-type Ig domains in some studies (Harpaz and Chothia, 1994; Freigang et al., 2000). All Ig domains of the IgSF CAMs have a core of two β-sheets facing each other and stabilized by an intra-chain disulfide bridge (Chothia et al., 1998; **Figure 2**). Ectodomains of IgSF CAMs may also contain fibronectin type III repeats, which are also present in ectodomains of some GPI-anchored IgSF CAMs, such as contactin-1, -2, -3 (**Figures 1, 2**).

In the human and murine genomes, genes coding for GPI-anchored IgSF CAMs include *NEGR1, opioid-binding cell adhesion molecule* (*OBCAM*), *neurotrimin* (*Ntm*), *limbic system-associated membrane protein* (*LAMP*), *IgLON5, contactin-1, -2, -3, -4, -5, -6, Thy-1*, and *carcinoembryonic antigen-related cell adhesion molecule* (*CEACAM)-5, -6, -7*, and *-8* (Williams and Gagnon, 1982; Oikawa et al., 1987; Yoshihara et al., 1994, 1995; Hachisuka et al., 1996; Ogawa et al., 1996; Funatsu et al., 1999; Itoh et al., 2008; Sabater et al., 2016) (**Table 1** and **Figure 1**). OBCAM, Ntm, LAMP, NEGR1, and IgLON5 constitute the IgLON family (Yamada et al., 2007; Hashimoto et al., 2009; Sanz et al., 2015). In addition, short isoforms of some transmembrane IgSF CAMs, such as the shortest isoform of the neural cell adhesion molecule (NCAM) with the molecular weight 120 kDa (NCAM120), are also GPI-anchored (Hemperly et al., 1986).

Glycosylphosphatidylinositol-anchoring of proteins to the plasma membranes is a highly conserved post-translational modification across all eukaryotes (Fujita and Kinoshita, 2012). The GPI anchor is a complex structure consisting of a phosphoethanolamine linker, glycan core, and phospholipid tail. Structural variations of the anchor are possible by the modification of phosphoinositol, glucosamine, and mannose residues within the glycan core (Paulick and Bertozzi, 2008; Fujita and Kinoshita, 2012). Application of phosphatidylinositol-specific phospholipase C (PI-PLC), an enzyme capable of cleaving the GPI anchor, induces removal of GPI-anchored IgSF CAMs from the cell surface, indicating that the GPI anchor is critical for the attachment of these proteins to the cellular membranes (Sanz et al., 2015). The complexity of the GPI anchor, however, suggests that it also plays a role in other multiple functions aside from membrane anchorage, including signal transduction, protein sorting, as well as the structure and function regulation of the GPI-anchored IgSF CAMs. In particular, the GPI anchor links these molecules to membrane microdomains that are insoluble in cold non-ionic detergents (Varma and Mayor, 1998; Fujita and Kinoshita, 2012). Such specialized membrane microdomains are referred to as lipid rafts and render GPI-anchored proteins resistant to cold non-ionic detergent extraction (Schroeder et al., 1994; Simons and Ikonen, 1997).

The ectodomains of GPI-anchored IgSF CAMs contain multiple glycosylation sites. For example, IgLON protein family members with three Ig domains contain six or seven *N*-glycosylation sites in their ectodomains (**Figure 1**; Pimenta et al., 1996; Itoh et al., 2008). NCAM120 is a prominent example of a glycosylated GPI-anchored IgSF CAM (Yoshihara et al., 1991), because similarly to transmembrane NCAM isoforms it can carry polysialic acid (PSA) (Dityatev et al., 2004).

Multiple subdomains in the extracellular domains of IgSF CAMs have been suggested to contain binding sites for interactions with other Ig domain-containing proteins (Brümmendorf and Rathjen, 1993). Indeed, Ig domains have been shown to play a key role in homophilic and heterophilic *trans-*interactions between IgSF CAMs, i.e., the interaction between two identical IgSF CAMs on membranes of adjacent cells, and the interaction of IgSF CAMs with other proteins in the extracellular environment (Reed et al., 2004; Walmod et al., 2004; Kulahin et al., 2011). In addition to mediating *trans*-interactions, IgSF CAMs bind in *cis*, i.e., laterally, to surface proteins present in the same cell surface plasma membrane (Held and Mariuzza, 2011). *Cis*-interactions can enhance the *trans*-interactions of IgSF CAMs and are also involved in signal transduction across the membrane (Soroka et al., 2003; Kiselyov et al., 2005). Similarly to transmembrane IgSF CAMs, GPI-anchored IgSF CAMs mediate homo- and heterophilic interactions. For example, LAMP, OBCAM, Ntm, Thy-1, CEACAM5, contactin-2, and NCAM120 bind homophilically *in trans* while CEACAM6 binds heterophilically *in trans* to CEACAM8 (Oikawa et al., 1991; Mahanthappa and Patterson, 1992; Rader et al., 1993; Zhou et al., 1993; Zhukareva and Levitt, 1995; Lodge et al., 2000; Taheri et al., 2000; Gil et al., 2002). Neurotractin (a chick homolog of human NEGR1) appears to be unique in the IgLON family in that it does not bind homophilically, but heterophilically binds to Ntm and LAMP (Marg et al., 1999), although a later study reported that mammalian NEGR1 was able to interact homophilically (Miyata et al., 2003a). GPI-anchored IgSF CAMs also interact in *cis*. For example, LAMP and OBCAM heterophilically interact in *cis* to create dimeric IgLONS (diglons) and formation of this complex changes the ability of both proteins to regulate neuronal development (Horstkorte et al., 1993; Ranheim et al., 1996; Reed et al., 2004; Held and Mariuzza, 2011).

### GPI-Anchored IgSF CAMs As Functional Receptors

The role for IgSF CAMs as functional receptors has been suggested by studies analyzing effects of antibodies against these molecules on neurite outgrowth. Early studies using antibodies against Thy-1 as a growth substrate showed that Thy-1 antibodies enhance regeneration of neurites in rat retinal ganglion neurons and promote survival of mouse cerebellar Purkinje cells (Leifer et al., 1984; Messer et al., 1984). Similarly, antibodies against Thy-1 promote neurite outgrowth in rat dorsal root ganglion (DRG) neurons when applied in the culture medium (Chen et al., 2005). Later, natural ligands of GPI-anchored IgSF CAMs have also been shown to induce neurite outgrowth changes. Retinal ganglion cells from Thy-1 knock-out mice show impaired neurite outgrowth over different substrates made of the proteins of the extracellular matrix, including fibronectin and collagen, and Thy-1 knock-out mice demonstrate abnormal retinal formation with thinner retinae (Simon et al., 1999). Thy-1 has also been identified as a receptor for αVβ3 integrin. Binding of integrins to Thy-1 at the neuronal cell surface induces signal transduction across the cell membrane resulting in inactivation of the c-Src protein tyrosine kinase, reduced neurite outgrowth, as well as neurite retraction (Herrera-Molina et al., 2012). In this study, neuron-derived Cath-a-differentiated (CAD) cells grown on a monolayer of DITNC1 astrocyte cells, which expressed αVβ3 and β3 integrins, had inhibited neurite outgrowth compared to CAD cells grown over a monolayer of DITNC1 cells treated with Thy-1-Fc protein, anti-β3 integrin antibodies, or transfected with siRNA against the β3 chain of the integrin. Neurite outgrowth inhibition in CAD cells on a substrate of αVβ3-Fc was abolished by silencing Thy-1 expression by shRNA. Furthermore, while αVβ3-Fc reduced dendritic length in primary cortical neurons, application of PI-PLC to cleave Thy-1 prior to the addition of αVβ3-Fc prevented the inhibition of dendrite outgrowth (Herrera-Molina et al., 2012).

Chondroitin sulfate E has been shown to activate contactin-1 to stimulate neurite outgrowth in primary mouse hippocampal neurons (Mikami et al., 2009). In addition to being a receptor to chondroitin sulfate E, contactin-1 binds to the second and third fibronectin type III (FNIII)-like domains of tenascin-R. Binding of tenascin-R to contactin-1 promotes neurite outgrowth (Norenberg et al., 1995), and induces formation of filopodia and lamellipodia along neurites (Zacharias, 2002). Contactin-1 is also a receptor for the receptor-type protein tyrosine phosphatase zeta (PTPRZ) (Peles et al., 1995; Bouyain and Watkins, 2010), and induces neurite outgrowth in chick tectal neurons in response to binding to PTPRZ (Peles et al., 1995). Recent work also showed that contactin-1 at the cell surface of hippocampal neurons binds in *trans* to contactin-associated transmembrane receptor 2 (CASPR2) (Rubio-Marrero et al., 2016). The physiological role of this interaction remains to be analyzed. The role for contactin-1 as a functional receptor in regulation of neuronal development is also supported by *in vivo* observations in contactin-1 knock-out mice. Granule cells are the major neuron population expressing contactin-1 in axons in the cerebellum. In wild-type mice, the parallel fibers of granule cells extend perpendicular to the dendritic arborizations of Purkinje cells. In contrast, the parallel fibers extend parallel to the plane of Purkinje cell dendritic branches in contactin-1 knock-out mice indicating misguidance of granule cell axon subpopulations (Berglund et al., 1999).

Contactin-2 has been shown to function as a receptor for neuronal cell adhesion molecule (NrCAM) but not for the neuron–glia cell adhesion molecule (NgCAM) in DRG and sympathetic ganglion neurons (Lustig et al., 1999). In these neurons, substrate-coated NgCAM and NrCAM, two L1 family CAMs, promote neurite outgrowth. Anti-contactin-2 Fab fragments do not affect the neurite outgrowth induced by NgCAM, but inhibit the NrCAM-dependent neurite outgrowth (Lustig et al., 1999). Substrate-coated NrCAM and NgCAM interact heterophilically in *trans* with contactin-2 at the cell surface of chick commissural axons and both molecules cooperate in the axonal guidance. However, this interaction is not involved in regulation of the axonal outgrowth (Fitzli et al., 2000). Knock down of contactin-2 with ex-ovo RNAi in the chick embryo affects guidance but not growth of axons of granule cells in the cerebellum (Baeriswyl and Stoeckli, 2008). Knock down of contactin-2 results in the failure of granule cells to extend their axons parallel to the pial surface of the cerebellum, creating an uneven molecular layer with decreased parallel fiber density. Since known binding partners of contactin-2, including NgCAM and NrCAM, are not expressed during granule cell axon extension, homophilic interactions of contactin-2 were proposed to be involved in axon guidance in these neurons (Baeriswyl and Stoeckli, 2008). Loss of contactin-2 in chick retinal neurons also impairs the ability of retinal neurons to contain their arbors within appropriate sublaminae (Yamagata and Sanes, 2012).

Glycosylphosphatidylinositol-anchored IgSF CAMs can also function as receptors when they are involved in homophilic binding. Cortical neurons grown on a substrate of recombinant OBCAM or LAMP demonstrate a dose-dependent increase in neurite outgrowth (Sanz et al., 2015). This neurite outgrowth-promoting activity of LAMP has been attributed to the first Ig domain within LAMP mediating homophilic interactions (Eagleson et al., 2003). Homophilic binding of Ntm also induces neurite growth in hippocampal neurons (Gil et al., 1998, 2002).

### GPI-Anchored IgSF CAMs As Signal Transducers and Membrane Domain Organizers

While GPI-anchored IgSF CAMs do not possess intracellular domains, they induce intracellular signaling and regulate formation of the functional membrane domains by interacting in *cis* with other transmembrane proteins. For example, activation of the intracellular signaling by Thy-1 antibodies is likely to be induced by cross-linking Thy-1 molecules and associated proteins, such as integrins (Kuroiwa et al., 2012).

Contactin-1 interacts in *cis* with CASPR (Peles et al., 1997). The CASPR/contactin-1 complex accumulates in paranodal junctions in myelinated axons during myelination of peripheral nerves (Rios et al., 2000). Contactin-1 is necessary to target CASPR to the synaptic membrane, because CASPR is synthesized but not targeted to the cell surface plasma membrane in the hippocampus of contactin-1-deficient mice (Rios et al., 2000; Murai et al., 2002). Contactin-1 also forms a complex with L1 and Fyn kinase in the mouse cerebellum, suggestive of the capability to transduce signals to intracellular proteins via L1 (Olive et al., 2002).

Contactin-2 binds in *cis* to L1. This interaction can be induced by homophilic *trans* interactions of contactin-2 resulting in the *cis* binding to L1 and L1-mediated ankyrin recruitment to the complex (Malhotra et al., 1998). Contactin-2 also binds in *cis* to NgCAM. This heterophilic *cis* interaction promotes neurite outgrowth, whereas heterophilic *trans* interaction between NgCAM and contactin-2 has no effect on neurite outgrowth (Buchstaller et al., 1996). Contactin-2 binds directly to the ectodomain of CASPR2 (Lu et al., 2016), and both proteins form a *cis* complex but are unable to form a *trans* complex. Despite this, contactin-2 is able to bind homophilically in *trans* to contactin-2 that has formed a *cis* complex with CASPR2 (Traka et al., 2003). The physiological significance of this interaction is illustrated by observations in contactin-2 and CASPR2 knock-out mice showing that contactin-2 is necessary for CASPR2 localization at juxtaparanodes in myelinated axons (Traka et al., 2003), whereas targeting of contactin-2 to juxtaparanodes depends on CASPR2 and both contactin-2 and CASPR2 are required for accumulation of voltage-gated potassium channels at the juxtaparanodes (Poliak et al., 2003).

Other contactins also associate in *cis* with various cell surface receptors. For example, contactin-5 forms a *cis* complex with amyloid precursor-like protein 1 (APLP1) on the presynaptic membrane (Shimoda et al., 2012). The second and third Ig domains of contactin-3, -4, -5, and -6 bind to receptor-type protein tyrosine phosphatase G (PTPRG) (Bouyain and Watkins, 2010) and contactin-3 and -6 associate in *cis* with PTPRG at the surface of mouse rod photoreceptor cells (Nikolaienko et al., 2016). Contactin-6 also interacts *in cis* with the Close Homolog of L1 (CHL1) and binds to and regulates the activity of the receptor-type protein tyrosine phosphatase α (PTPRα) (Ye et al., 2008).

Glycosylphosphatidylinositol-anchored IgSF CAMs also interact with the intracellular enzymes and cytoskeleton via lipids. NCAM120 co-localizes and associates with the membrane-cytoskeleton linker protein spectrin in transfected CHO cells and mouse hippocampal neurons, and this association is lost after disruption of the lipid rafts (Leshchyns’ka et al., 2003). Overexpression of NCAM120 in cultured hippocampal neurons from NCAM knock-out mice, however, is not sufficient to induce neurite outgrowth in response to recombinant extracellular domain of NCAM (Niethammer et al., 2002). The interaction with spectrin may, however, be important in glial cells where NCAM120 is enriched. Contactin-2 associates with ganglioside GD3 in cerebellar neurons, and clustering of this complex induces activation of the Src family kinase Lyn (Kasahara et al., 2000, 2002). A recent study showing that NEGR1 interacts with Niemann-Pick disease Type C2 (NPC2) protein and functions in cholesterol transport (Kim et al., 2017) suggests that GPI-anchored IgSF CAMs may also be involved in regulation of the lipid composition of the plasma membrane.

### GPI-Anchored IgSF CAMs As Functional Ligands

Glycosylphosphatidylinositol-anchored IgSF CAMs also function as ligands for other cell surface receptors in neurons and other cells. Early work on contactin-1 has demonstrated that neurite outgrowth in DRG neurons grown on CHO cells transfected with contactin-1 is increased (Gennarini et al., 1989, 1991). Further work showed that contactin-1 exerts dual cell-specific effects on neurite outgrowth by inhibiting neurite outgrowth in cerebellar granule cells and stimulating neurite outgrowth in sensory neurons, whereas it does not affect hippocampal neurons (Gennarini et al., 1991; Buttiglione et al., 1996), suggesting that contactin-1 activates different receptors expressed by these cells (Buttiglione et al., 1996). Among neuronal receptors for contactin-1 are members of the L1 family. NrCAM and NgCAM have been shown to interact heterophilically with contactin-1 (Morales et al., 1993). However, despite having shown that contactin-1 interacts with NrCAM and NgCAM, only NrCAM was found to enhance the outgrowth of chick retinal neurons (Treubert and Brümmendorf, 1998). Tectal cells adhere to and extend neurites on a substrate of contactin-1 and this effect is blocked by the application of Fab fragments against NrCAM but not NgCAM (Morales et al., 1993). While these experiments indicate that the interaction between contactin-1 and NrCAM induces neurite outgrowth, receptors mediating the inhibitory effects of contactin-1 on neurite outgrowth and the role that the interaction between contactin-1 and NgCAM plays remain to be determined. Contactin-1 was also identified to be a ligand of Notch in oligodendrocytes being involved in the signaling pathway of oligodendrocyte maturation (Hu et al., 2003). The role for contactin-1 as a functional ligand is also supported by *in vivo* observations. Contactin-1 is not detected in dendrites of granule cells (Faivre-Sarrailh et al., 1992). However, development of the dendrites is affected in contactin-1 knock-out mice resulting in a significant reduction of granule cell postsynaptic area (Berglund et al., 1999), suggesting that contactin-1 expressed on other cells acts as a functional ligand to regulate dendrite formation.

Contactin-2 used as a substrate induces neurite outgrowth in rat and chick DRG neurons (Furley et al., 1990; Stoeckli et al., 1991) and in rat and mouse cerebellar neurons (Kasahara et al., 2002; Wang et al., 2011). Removal of contactin-2 by PI-PLC from DRG neurons cultured on a substrate of contactin-2 does not affect the effect of contactin-2 substrate on neurite outgrowth (Felsenfeld et al., 1994) indicating that homophilic interactions of contactin-2 are not involved. Contactin-2-dependent neurite outgrowth is blocked by Fab fragments against L1 (Kuhn et al., 1991; Stoeckli et al., 1991; Felsenfeld et al., 1994; Wang et al., 2011) and β1 integrin (Felsenfeld et al., 1994), indicating that L1 and integrins are contactin-2 receptors, which promote neurite outgrowth. Contactin-2 was also shown to be a ligand of amyloid precursor protein (APP). Binding of contactin-2 to APP triggers cleavage of APP resulting in the release of its intracellular domain, which negatively modulates neurogenesis (Ma et al., 2008).

Neurite outgrowth in rat hippocampal neurons is enhanced when they are grown on substrate-coated recombinant contactin-3 and -4 (Yoshihara et al., 1994, 1995), or on HEK293 cells transfected with contactin-4, -5, and -6 (Bouyain and Watkins, 2010; Mercati et al., 2013), indicating that other CAMs of the contactin family can also function as *trans* ligands. Contactin-3 and -6 associate with PTPRG not only in *cis* but also in *trans* when expressed on the surfaces of apposing cells (Nikolaienko et al., 2016). Hence, PTPRG is likely to be a neuronal receptor for these CAMs of the contactin family. However, contactin-4, -5, and -6 display identical binding to PTPRG but differentially promote neurite outgrowth and branching at distinct developmental stages (Mercati et al., 2013), suggesting that other receptors are also involved. These receptors remain to be identified in future work.

*Trans*-interactions have also been reported for Thy-1 presented as a ligand. Thy-1 was identified in a neurite outgrowth-promoting complex containing also laminin and a heparin sulfate proteoglycan (Greenspan and O’brien, 1989). Thy-1 expressed at the neuronal cell surface functions as a ligand for αVβ3 integrin at the cell surface of astrocytes (Hermosilla et al., 2008). This interaction leads to integrin clustering, tyrosine phosphorylation of focal adhesion kinase (FAK) and p130Cas, activation of RhoA and p160ROCK, recruitment of paxillin, vinculin, and FAK to focal contacts resulting in formation of focal adhesion and stress fibers in rat astrocytes (Leyton et al., 2001; Avalos et al., 2002, 2004).

Cell adhesion molecules of the IgLON family NEGR1 and Ntm are constitutively shed from the cell surface and create a growth permissive substrate. Inhibition of their shedding by a pan-metalloproteinase inhibitor (BB-94) inhibits neurite outgrowth in cortical neurons and IgLON CAMs accumulate at the cell surface, whereas an increase in shedding by PI-PLC promotes neurite outgrowth (Sanz et al., 2015). A recent report showing that soluble NEGR1 promotes neuronal arborization in FGFR2- and ERK1/2-dependent manner (Pischedda and Piccoli, 2015) suggests that FGFR2 is one of the receptors for NEGR1 at the neuronal cell surface. The shedding of IgLON CAMs not only provides a growth permissive substrate but also renders cortical neurons grown on the IgLON substrate insensitive to the growth inhibitory effects of BB-94 suggesting that shed IgLON CAMs mitigate inhibitory signals transduced by a NEGR1- or Ntm-containing complex at the neuronal cell surface (Sanz et al., 2015).

Another member of IgLON family, LAMP, inhibits the neurite outgrowth in DRG neurons by heterophilically binding to Ntm expressed in these neurons (Gil et al., 2002). The second Ig domain of LAMP, which is not involved in homophilic interactions, harbors the outgrowth inhibiting activity (Eagleson et al., 2003). Soluble recombinant Ntm induces neurite outgrowth in DRG neurons. This effect is also observed after removal of Ntm from the cell surface of these neurons by PI-PLC indicating that it is mediated by heterophilic interactions of Ntm. In contrast, Ntm inhibits neurite outgrowth in SCG neurons. These neurons do not express Ntm, and therefore Ntm’s effects are also mediated by heterophilic interactions (Gil et al., 1998, 2002).

### GPI-Anchored IgSF CAMs Are Present in Synapses in Neurons

The presence of multiple GPI-anchored IgSF CAMs in synapses was first suggested by biochemical analysis of synaptic terminals, synaptosomes, isolated from the brain tissue. Early work with Thy-1 antigen showed that Thy-1 was present in synaptosomes isolated from the mouse brain with later work showing that Thy-1 is a component of large dense core and small clear vesicles of PC12 cells which are similar to neuronal synaptic vesicles (Stohl and Gonatas, 1977; Jeng et al., 1998). Later studies showed the presence of different GPI-anchored IgSF CAMs in synaptosomes, including contactin-1 and contactin-2 (Murai et al., 2002; Bakkaloglu et al., 2008).

Immunoelectron microscopic analysis of contactin-1 localization revealed that depending on the type of synapse, contactin-1 is localized to either the pre- or post-synaptic membranes (Faivre-Sarrailh et al., 1992). For example, in the mouse cerebellum contactin-1 is localized pre-synaptically in synapses between parallel fibers of granule cells and dendritic spines of Purkinje cells and in synapses between mossy fiber terminals and granule cell dendrites, and is localized post-synaptically in synapses formed on Golgi cell dendrites (Faivre-Sarrailh et al., 1992). In the hippocampal CA1 region, contactin-1 is distributed at the surface of pyramidal cell dendrites, dendritic spine heads, and post-synaptic densities, and is also present in biochemically isolated post-synaptic density fractions (Murai et al., 2002). Contactin-2 has also been shown to localize to synaptic plasma membranes isolated from rat forebrain (Bakkaloglu et al., 2008).

In contrast to contactin-1, Ntm has been shown to accumulate both pre- and post-synaptically in synapses between parallel fibers of granule cells and dendritic spines of Purkinje cells and in synapses between mossy fiber terminals and granule cell dendrites but was not present in inhibitory synapses made by stellate or basket cells (Chen et al., 2001). LAMP is expressed pre- and post-synaptically in synapses in the developing lateral septum, but is detected only post-synaptically in synapses formed on granule cells of the dentate gyrus in adult hippocampus (Zacco et al., 1990).

Electron microscopic immunohistochemistry has demonstrated that NEGR1 is present at high levels in post-synaptic densities and at lower levels pre-synaptically in synapses along dendrites and on somata of neurons in the cerebral cortex and hippocampal CA3 region of adult rats (Miyata et al., 2003a). OBCAM shows similar ultrastructural distribution as NEGR1 with OBCAM immunoreactivity limited to postsynaptic densities of dendritic and somatic synapses in the cerebral cortex and hippocampal CA3 region of adult rats (Miyata et al., 2003a).

Several studies indicate that GPI-anchored IgSF CAMs are also present in synaptic organelles. Biochemical analysis of Thy-1 in the rat brain showed that it is targeted to small synaptic vesicles (SSVs) and large dense core vesicles (LDCVs) (Jeng et al., 1998). OBCAM is present in neurosecretory granules in neurites of hypothalamic magnocellular neurons (Miyata et al., 2003b). Mass spectrometry analysis of synaptic vesicles also identified NEGR1, OBCAM, Ntm, Thy-1, LAMP, and contactin-1 as components of synaptic vesicles (Takamori et al., 2006).

### Role of the GPI-Anchored IgSF CAMs in Synapse Formation Regulation

Protein expression and localization of the GPI-anchored IgSF CAMs is developmentally regulated. Levels of Thy-1 strongly increase from postnatal day 14, whereas levels of NEGR1, OBCAM, and contactin-1 gradually increase during development and reach the highest level at 4 weeks after birth in the cerebral cortex, diencephalon, hippocampus, and cerebellum (Miyata et al., 2003a). Ntm levels gradually increase in the forebrain during development reaching the plateau at postnatal day 7 and then decline in adults (Struyk et al., 1995). Ntm levels are also increased in the molecular layer and the internal granular layer of the cerebellum during the period of synaptogenesis and reduce shortly after the active period of synaptogenesis ends, but remain high at synaptic contacts (Chen et al., 2001). Levels of NCAM and particularly NCAM120 increase in chick cornea and corneal nerves during corneal innervation (Mao et al., 2012). High expression of the GPI-anchored IgSF CAMs at the time of active synaptogenesis and their synaptic localization suggests that they play a role in synapse formation.

The role of the GPI-anchored IgSF CAMs in synapse formation is further indicated by studies showing that disruption of their functions or increase in their levels in neurons affect synaptogenesis. Overexpression of OBCAM in hippocampal neurons increases numbers of synapses along dendrites of transfected neurons (Hashimoto et al., 2009), whereas disruption of OBCAM functions using antibodies or by suppressing its expression using the antisense oligodeoxynucleotide results in impaired formation of synapses on dendrites of hippocampal neurons indicating that OBCAM promotes synapse formation (Yamada et al., 2007). OBCAM expression has been observed to be higher during early postnatal development and it decreases over time suggesting that OBCAM is active in the regulation of synapse formation (Li et al., 2006; Yamada et al., 2007). An increase in synapse formation has also been observed in cultured hippocampal neurons overexpressing LAMP (Hashimoto et al., 2009) and NCAM120 (Dityatev et al., 2004). Interestingly, overexpression of NEGR1 decreases numbers of synapses formed on dendrites of hippocampal neurons (Hashimoto et al., 2009). Thus, different GPI-anchored IgSF CAMs not only promote, but can also reduce synaptogenesis.

The molecular mechanisms of synaptogenesis regulation by the GPI-anchored IgSF CAMs remain poorly understood. Interestingly, ablation of NCAM expression in GABAergic basket interneurons in the postnatal mouse cortex results in impaired maturation of perisomatic synapses formed by these neurons, and this phenotype is rescued by NCAM120 (Chattopadhyaya et al., 2013). The NCAM120-dependent maturation of synapses is inhibited by a dominant-negative form of Fyn kinase (Chattopadhyaya et al., 2013) indicating that GPI-anchored IgSF CAMs regulate synapse formation not only via changes in cell adhesion but also by activating intracellular signaling.

### GPI-Anchored IgSF CAMs in Regulation of Synaptic Plasticity

Recent reports indicate that GPI-anchored IgSF CAMs also play a role in synaptic plasticity. Constitutively contactin-1-deficient mice show decreased paired pulse facilitation (PPF). Long-term potentiation (LTP) in the CA1 region of the hippocampus of these mice is normal, whereas long-term depression (LTD) is impaired (Murai et al., 2002). In contactin-1 transgenic mice generated to induce overexpression of full-length contactin-1 under control of the human contactin-2 promoter, PPF is not changed at 5 and 12 months of age indicating that the short-term plasticity is not altered by contactin-1 overexpression. However, LTP in the CA1 region of the hippocampus is increased in contactin-1-overexpressing mice at 12 months of age despite no change in LTP in contactin-1 transgenic mice at 5 months of age (Puzzo et al., 2013). Further analysis of LTP in the CA1 region of the hippocampus in older animals showed that LTP is impaired in 24-month-old wild-type mice when compared to 3–5-month-old wild-type animals. However, the age-dependent decline in LTP was slower in mice overexpressing contactin-1. Hence, contactin-1 is likely to play a role in maintaining synaptic plasticity in the adult brain during aging (Puzzo et al., 2013, 2015). Synaptic transmission is impaired in LAMP knock-out mice with a reduction in LTP in the CA1 region of the hippocampus (Qiu et al., 2010). LTP in the CA1 region of the hippocampus in Thy-1 knock-out mice was normal although LTP was absent in the dentate gyrus of these mice (Nosten-Bertrand et al., 1996). These observations indicate that GPI-anchored IgSF CAMs are involved in regulation of the different forms of synaptic plasticity in brain region-dependent manner.

### GPI-Anchored IgSF CAMs in Regulation of Learning and Behavior

Glycosylphosphatidylinositol-anchored IgSF CAMs are also involved in regulation of learning and behavior. Thy-1 knock-out mice have been found to fail in observing social cues to select food that had been socially cued (Mayeux-Portas et al., 2000). It was therefore proposed that the loss of Thy-1 while not fatal is still under evolutionary pressure as the inability to select cued (and therefore safe) food would quickly push for the conservation of Thy-1. Spatial learning as assessed by the Morris water maze test was not affected in Thy-1 knock-out mice (Nosten-Bertrand et al., 1996).

Mice deficient for LAMP exhibit heightened reactivity to novelty, lower anxiety, and lower sensitivity to stressful environment (Catania et al., 2008; Innos et al., 2011, 2012). LAMP deficiency in mice also results in impaired spatial learning as indicated by increased time LAMP-deficient mice need to locate the underwater platform in the Morris water maze (Qiu et al., 2010). The role for LAMP in regulation of behavior is further suggested by observations showing that LAMP expression levels are increased in the hippocampus of mice exposed to the enriched environment (Heinla et al., 2015).

The link between changes in contactin-1 expression and age-dependent learning impairments has been demonstrated in studies showing that the age-dependent decline in contactin-1 levels in wild-type mice correlates with a higher latency in finding the hidden platform in the Morris water maze (Palmeri et al., 2013; Puzzo et al., 2015). The age-dependent increase in the time required to find the hidden platform in the Morris water maze is reduced in transgenic contactin-1-overexpressing mice (Puzzo et al., 2015). Furthermore, while old wild-type mice spend equal amounts of time exploring the novel and familiar objects in the novel object recognition test analyzing recognition memory, the old contactin-1-overexpressing mice spend more time exploring the novel object (Puzzo et al., 2015).

### GPI-Anchored IgSF CAMs in Brain Disorders

Several observations indicate that contactin-2 may play a role in the onset of epilepsy. A homozygous single base pair deletion (c.503_503delG) of contactin-2 was identified to be present in individuals affected with autosomal recessive cortical myoclonic tremor and epilepsy in a consanguineous Egyptian family (Stogmann et al., 2013). The role of contactin-2 in epilepsy is further suggested by studies in mice showing that while gross brain morphology of contactin-2 null mice appears to be indistinguishable from their wild-type littermates, contactin-2 null mice display spontaneous episodes of seizures despite demonstrating normal behavior and are more sensitive to convulsant stimuli than their wild-type littermates (Fukamauchi et al., 2001).

Genome-wide analysis of copy number variations in autism spectrum disorder (ASD) patients identified a trend for the contactin gene family (*contactin-4, -5, -6*) to be associated with ASD (Van Daalen et al., 2011; Nava et al., 2014; Poot, 2014). The identification of multiple contactin CAMs in ASD has led to the suggestion that genetic interactions between contactins are involved in different degrees of ASD (Poot, 2014).

Autoantibodies against contactin-1 were shown to be present in a subset of patients suffering from chronic inflammatory demyelinating polyneuropathy (CIDP) (Querol et al., 2013), suggesting that contactin-1 is involved in neuromyopathies. Chronic passive transfer of anti-contactin-1 IgG4 in Lewis rats results in progressive motor deterioration (Manso et al., 2016), indicating that antibodies against contactin-1 are pathogenic in CIDP. A study on a consanguineous family with a homozygous contactin-1 mutation presenting with lethal congenital myopathy also supports the role contactin-1 may have in peripheral neuromyopathies (Compton et al., 2008).

Increased levels of NCAM120 were found in the cerebrospinal fluid (CSF) of patients suffering from bipolar disorder and depression suggesting that NCAM120 may be involved in mood disorders (Poltorak et al., 1996).

Patient case studies found that deletions of 1p31.1 to 1p31.3 containing the *NEGR1* gene present with developmental co-ordination disorder, attention deficit/hyperactivity disorder, learning disability, as well as delayed speech and language development (Gillberg and FitzPatrick, 2010; Tassano et al., 2015). A genome-wide copy number scan identified *NEGR1* to be one of five new candidate genes involved in dyslexia (Veerappa et al., 2013). A case study on two siblings with interstitial microdeletion of 1p31.1 involving only *NEGR1* presented with learning and behavioral problems, hypotonia, hypermobility, scoliosis, and aortic root dilation (Genovese et al., 2015). Further suggestive of the role NEGR1 has in brain disorders, NEGR1 is also elevated in the CSF of bipolar and depressed patients (Maccarrone et al., 2013). In Dark Agouti rats, NEGR1 is upregulated in response to venlafaxine (VLX), a serotonin and noradrenaline reuptake inhibitor used to treat major depressive disorder (MDD), suggesting that NEGR1 contributes to the VLX effect in MDD possibly by contributing to the establishment of new neuronal connections and changes in synaptic plasticity (Tamási et al., 2014).

A study on male contemplated suicide identified LAMP SNPs to be associated with suicide (Must et al., 2008). However, it is important to note that after multiple correction tests the association did not maintain statistical significance leading the authors to suggest that LAMP may play a role in suicidal behavior but more work is required to confirm their initial findings. Genotyping showed that four SNPs (rs1461131, rs4831089, rs16824691, and rs9874470) of LAMP were significantly associated with MDD (Koido et al., 2012). In addition, LAMP expression was significantly increased in the dorsolateral prefrontal cortex of schizophrenic and bipolar disorder patients (Behan et al., 2009).

In a study on late-onset Alzheimer’s disease, four SNPs (rs1629316, rs1547897, rs11222931, and rs11222932) in intron 1 of the Ntm gene (11q25) and one SNP (rs11223225) in intron 1 of the OBCAM gene (located on the same chromosome as Ntm < 80 kb apart) have been found to be associated with late-onset Alzheimer’s disease (Liu et al., 2007). Genome-linkage studies in two independent Dutch populations indicate that depression is also associated with a locus on chromosome 11q25 suggesting a link to OBCAM (Schol-Gelok et al., 2010). In a study of schizophrenia in Thai populations, four SNPs (rs3016384, rs1784519, rs1894193, and rs1939498) of OBCAM have been identified to be linked to schizophrenia (Panichareon et al., 2012). An earlier study also identified OBCAM to be implicated in schizophrenia; however, the association was nominally significant (O’Donovan et al., 2008). Genome-wide analysis of aggressiveness in attention deficit hyperactivity disorder (ADHD) found that one of two significant loci associated with aggressiveness in ADHD was within the Ntm gene (Brevik et al., 2016). Additionally, Ntm has also been identified to be associated with intelligence as demonstrated by a family-based association study (Pan et al., 2011).

### Conclusion and Future Directions

Glycosylphosphatidylinositol-anchored IgSF CAMs play important roles in regulation of neuronal development, synapse formation and function, learning, and behavior. Previous research indicates that in addition to mediating adhesive interactions, these molecules induce intracellular signaling by binding to other cell surface receptors, regulating their levels and functions, and assembling membrane microdomains.

Further research is, however, needed to characterize the whole repertoire of the interactions of GPI-anchored IgSF CAMs in developing and mature neurons, and in synapses to fully understand the role these molecules play in the developing and mature nervous system and molecular mechanisms involved. While several neurite outgrowth-promoting receptors for GPI-anchored IgSF CAMs have been described, observations showing that even relatively well-characterized contactin-1 not only promotes but also inhibits neurite outgrowth in a neuron-specific manner suggest that there are still other receptors that remain to be identified. How the repertoire of molecular interactions involving GPI-anchored IgSF CAMs changes during neuronal development remains also poorly understood. The roles that these interactions play in regulating synapse formation and function are mostly unknown. It is possible that GPI-anchored IgSF CAMs promote synapse formation by forming homophilic adhesive bonds connecting pre- and post-synaptic membranes. However, these molecules are often asymmetrically expressed in synapses and are found either pre- or post-synaptically. This observation suggests that GPI-anchored IgSF CAMs regulate synapse formation by heterophilically interacting with other receptors or CAMs in synaptic membranes. Further characterization of the synaptic interactions of GPI-anchored IgSF CAMs is necessary to understand molecular mechanisms activated by these molecules during synapse formation. It may also help to understand the role that GPI-anchored IgSF CAMs play in synaptic plasticity suggested by abnormalities in mice with altered expression of these molecules, and to characterize the signaling pathways regulated by GPI-anchored IgSF CAMs in synapses.

Further analysis of the post-translational modifications of GPI-anchored IgSF CAMs is necessary to understand mechanisms of the regulation of the functions of these molecules. Shedding of the IgLON family members was shown to play an important role during neuronal development. The role that proteolysis of GPI-anchored IgSF CAMs plays in regulation of synapse numbers and remodeling during synaptic plasticity remains to be investigated. Regulation of the homophilic and heterophilic interactions of GPI-anchored IgSF CAMs by glycosylation of the multiple sites within their ectodomains is also an intriguing possibility, which remains to be analyzed. Whether GPI anchor-mediated interactions with lipids play a role in the transport of GPI-anchored IgSF CAMs, their sorting to lipid rafts, microdomain assembly, and signal transduction also remains to be investigated.

Genetic association studies in humans have started to illuminate the role of GPI-anchored IgSF CAMs in brain disorders. Biochemical analyses of the changes in levels and synaptic targeting of GPI-anchored IgSF CAMs in postmortem human brain tissue and in animal models are necessary to corroborate these findings and may reveal yet unknown roles of these molecules in brain disorders. Future research analyzing molecular mechanisms of GPI-anchored IgSF CAM function and regulation will help to understand the molecular mechanisms of brain disorders linked to abnormal expression or function of these molecules, and may pave the way for development of new treatments of these disorders.

## Author Contributions

RT, IL, and VS were involved in analyzing the literature and writing the manuscript.

## Conflict of Interest Statement

The authors declare that the research was conducted in the absence of any commercial or financial relationships that could be construed as a potential conflict of interest.

## References

[B1] Avalos A. M. Arthur W. T. Schneider P. Quest A. F. G. Burridge K. Leyton L. (2004). Aggregation of integrins and RhoA activation are required for Thy-1-induced morphological changes in astrocytes. *J. Biol. Chem.* 279 39139–39145. 10.1074/jbc.M403439200 15220352

[B2] Avalos A. M. Labra C. V. Quest A. F. G. Leyton L. (2002). Signaling triggered by Thy-1 interaction with β3 integrin on astrocytes is an essential step towards unraveling neuronal Thy-1 function. *Biol. Res.* 35 231–238. 10.4067/S0716-97602002000200015 12415741

[B3] Baeriswyl T. Stoeckli E. T. (2008). Axonin-1/TAG-1 is required for pathfinding of granule cell axons in the developing cerebellum. *Neural Dev.* 3:7. 10.1186/1749-8104-3-7 18346270 PMC2322981

[B4] Bakkaloglu B. O’Roak B. J. Louvi A. Gupta A. R. Abelson J. F. Morgan T. M. (2008). Molecular cytogenetic analysis and resequencing of contactin associated protein-like 2 in autism spectrum disorders. *Am. J. Hum. Genet.* 82 165–173. 10.1016/j.ajhg.2007.09.017 18179895 PMC2253974

[B5] Barclay A. N. (2003). Membrane proteins with immunoglobulin-like domains - A master superfamily of interaction molecules. *Semin. Immunol.* 15 215–223. 10.1016/S1044-5323(03)00047-2 14690046

[B6] Behan Á. Byrne C. Dunn M. J. Cagney G. Cotter D. R. (2009). Proteomic analysis of membrane microdomain-associated proteins in the dorsolateral prefrontal cortex in schizophrenia and bipolar disorder reveals alterations in LAMP, STXBP1 and BASP1 protein expression. *Mol. Psychiatry* 14 601–613. 10.1038/mp.2008.7 18268500

[B7] Berglund E. O. Murai K. K. Fredette B. Sekerková G. Marturano B. Weber L. (1999). Ataxia and abnormal cerebellar microorganization in mice with ablated contactin gene expression. *Neuron* 24 739–750. 10.1016/S0896-6273(00)81126-5 10595523

[B8] Bouyain S. Watkins D. J. (2010). The protein tyrosine phosphatases PTPRZ and PTPRG bind to distinct members of the contactin family of neural recognition molecules. *Proc. Natl. Acad. Sci. U.S.A.* 107 2443–2448. 10.1073/pnas.0911235107 20133774 PMC2823867

[B9] Brevik E. J. van Donkelaar M. M. J. Weber H. Sánchez-Mora C. Jacob C. Rivero O. (2016). Genome-wide analyses of aggressiveness in attention-deficit hyperactivity disorder. *Am. J. Med. Genet. Part B Neuropsychiatr. Genet.* 171 733–747. 10.1002/ajmg.b.32434 27021288 PMC5071721

[B10] Brümmendorf T. Michael Wolff J. Frank R. Rathjen F. G. (1989). Neural cell recognition molecule F11: homology with fibronectin type III and immunoglobulin type C domains. *Neuron* 2 1351–1361. 10.1016/0896-6273(89)90073-1 2627374

[B11] Brümmendorf T. Rathjen F. G. (1993). Axonal glycoproteins with immunoglobulin- and fibronectin type III-related domains in vertebrates: structural features, binding activities, and signal transduction. *J. Neurochem.* 61 1207–1219. 10.1111/j.1471-4159.1993.tb13611.x 8376980

[B12] Buchstaller A. Kunz S. Berger P. Kunz B. Ziegler U. Rader C. (1996). Cell adhesion molecules NgCAM and axonin-1 form heterodimers in the neuronal membrane and cooperate in neurite outgrowth promotion. *J. Cell Biol.* 135 1593–1607. 10.1083/jcb.135.6.1593 8978825 PMC2133975

[B13] Buckley C. D. Rainger G. E. Bradfield P. F. Nash G. B. Simmons D. L. (1998). Cell adhesion: more than just glue (Review). *Mol. Membr. Biol.* 15 167–176. 10.3109/09687689709044318 10087503

[B14] Buttiglione M. Revest J. M. Rougon G. Faivre-Sarrailh C. (1996). F3 neuronal adhesion molecule controls outgrowth and fasciculation of cerebellar granule cell neurites: a cell-type-specific effect mediated by the Ig-like domains. *Mol. Cell. Neurosci.* 8 53–69. 10.1006/mcne.1996.0043 8923455

[B15] Catania E. H. Pimenta A. Levitt P. (2008). Genetic deletion of *Lsamp* causes exaggerated behavioral activation in novel environments. *Behav. Brain Res.* 188 380–390. 10.1016/j.bbr.2007.11.022 18199495 PMC2275759

[B16] Chattopadhyaya B. Baho E. Huang Z. J. Schachner M. Di Cristo G. (2013). Neural cell adhesion molecule-mediated Fyn activation promotes GABAergic synapse maturation in postnatal mouse cortex. *J. Neurosci.* 33 5957–5968. 10.1523/JNEUROSCI.1306-12.2013 23554477 PMC6618917

[B17] Chen C. H. Wang S. M. Yang S. H. Jeng C. J. (2005). Role of Thy-1 in in vivo and in vitro neural development and regeneration of dorsal root ganglionic neurons. *J. Cell. Biochem.* 94 684–694. 10.1002/jcb.20341 15547945

[B18] Chen S. Gil O. Ren Y. Q. Zanazzi G. Salzer J. L. Hillman D. E. (2001). Neurotrimin expression during cerebellar development suggests roles in axon fasciculation and synaptogenesis. *J. Neurocytol.* 30 927–937. 10.1023/A:1020673318536 12373100

[B19] Chothia C. Gelfand I. Kister A. (1998). Structural determinants in the sequences of immunoglobulin variable domain. *J. Mol. Biol.* 278 457–479. 10.1006/jmbi.1998.1653 9571064

[B20] Chothia C. Jones E. Y. (1997). The molecular structure of cell adhesion molecules. *Annu. Rev. Biochem.* 66 823–862. 10.1146/annurev.biochem.66.1.823 9242926

[B21] Compton A. G. Albrecht D. E. Seto J. T. Cooper S. T. Ilkovski B. Jones K. J. (2008). Mutations in contactin-1, a neural adhesion and neuromuscular junction protein, cause a familial form of lethal congenital myopathy. *Am. J. Hum. Genet.* 83 714–724. 10.1016/j.ajhg.2008.10.022 19026398 PMC2668069

[B22] Dityatev A. Dityateva G. Sytnyk V. Delling M. Toni N. Nikonenko I. (2004). Polysialylated neural cell adhesion molecule promotes remodeling and formation of hippocampal synapses. *J. Neurosci.* 24 9372–9382. 10.1523/JNEUROSCI.1702-04.2004 15496673 PMC6730092

[B23] Eagleson K. L. Pimenta A. F. Burns M. M. Fairfull L. D. Cornuet P. K. Zhang L. (2003). Distinct domains of the limbic system-associated membrane protein (LAMP) mediate discrete effects on neurite outgrowth. *Mol. Cell. Neurosci.* 24 725–740. 10.1016/S1044-7431(03)00237-9 14664821

[B24] Faivre-Sarrailh C. Gennarini G. Goridis C. Rougon G. (1992). F3/F11 cell surface molecule expression in the developing mouse cerebellum is polarized at synaptic sites and within granule cells. *J. Neurosci.* 12 257–267. 1729438 10.1523/JNEUROSCI.12-01-00257.1992 PMC6575693

[B25] Felsenfeld D. P. Hynes M. A. Skoler K. M. Furley A. J. Jessell T. M. (1994). TAG-1 can mediate homophilic binding, but neurite outgrowth on TAG-1 requires an L1-like molecule and β1 integrins. *Neuron* 12 675–690. 10.1016/0896-6273(94)90222-4 7512353

[B26] Fitzli D. Stoeckli E. T. Kunz S. Siribour K. Rader C. Kunz B. (2000). A direct interaction of axonin-1 with NgCAM-related cell adhesion molecule (NrCAM) results in guidance, but not growth of commissural axons. *J. Cell Biol.* 149 951–968. 10.1083/jcb.149.4.951 10811834 PMC2174557

[B27] Freigang J. Proba K. Leder L. Diederichs K. Sonderegger P. Welte W. (2000). The crystal structure of the ligand binding module of axonin-1/TAG-1 suggests a zipper mechanism for neural cell adhesion. *Cell* 101 425–433. 10.1016/S0092-8674(00)80852-1 10830169

[B28] Fujita M. Kinoshita T. (2012). GPI-anchor remodeling: potential functions of GPI-anchors in intracellular trafficking and membrane dynamics. *Biochim. Biophys. Acta* 1821 1050–1058. 10.1016/j.bbalip.2012.01.004 22265715

[B29] Fukamauchi F. Aihara O. Wang Y. J. Akasaka K. Takeda Y. Horie M. (2001). TAG-1-deficient mice have marked elevation of adenosine A1 receptors in the hippocampus. *Biochem. Biophys. Res. Commun.* 281 220–226. 10.1006/bbrc.2001.4334 11178983

[B30] Funatsu N. Miyata S. Kumanogoh H. Shigeta M. Hamada K. Endo Y. (1999). Characterization of a novel rat brain glycosylphosphatidylinositol-anchored Protein (Kilon), a member of the IgLON Cell adhesion molecule family. *J. Biol. Chem.* 274 8224–8230. 10.1074/jbc.274.12.8224 10075727

[B31] Furley A. J. Morton S. B. Manalo D. Karagogeos D. Dodd J. Jessell T. M. (1990). The axonal glycoprotein TAG-1 is an immunoglobulin superfamily member with neurite outgrowth-promoting activity. *Cell* 61 157–170. 10.1016/0092-8674(90)90223-2 2317872

[B32] Gennarini G. Cibelli G. Rougon G. Mattei M. G. Goridis C. (1989). The mouse neuronal cell surface protein F3: a phosphatidylinositol-anchored member of the immunoglobulin superfamily related to chicken contactin. *J. Cell Biol.* 109 775–788. 10.1083/jcb.109.2.775 2474555 PMC2115732

[B33] Gennarini G. Durbec P. Boned A. Rougon G. Goridis C. (1991). Transfected F3/F11 neuronal cell surface protein mediates intercellular adhesion and promotes neurite outgrowth. *Neuron* 6 595–606. 10.1016/0896-6273(91)90062-5 2015094

[B34] Genovese A. Cox D. M. Butler M. G. (2015). Partial deletion of chromosome 1p31.1 including only the neuronal growth regulator 1 gene in two siblings. *J. Pediatr. Genet.* 4 23–28. 10.1055/s-0035-1554977 27617112 PMC4906414

[B35] Gil O. D. Zanazzi G. Struyk A. F. Salzer J. L. (1998). Neurotrimin mediates bifunctional effects on neurite outgrowth via homophilic and heterophilic interactions. *J. Neurosci.* 18 9312–9325. 9801370 10.1523/JNEUROSCI.18-22-09312.1998 PMC6792904

[B36] Gil O. D. Zhang L. Chen S. Ren Y. Q. Pimenta A. Zanazzi G. (2002). Complementary expression and heterophilic interactions between IgLON family members neurotrimin and LAMP. *J. Neurobiol.* 51 190–204. 10.1002/neu.10050 11984841

[B37] Gillberg C. FitzPatrick D. (2010). Case report: further evidence for a recognisable syndrome caused by deletion of 1p31. *Adv. Clin. Neurosci. Rehabil.* 10 16–17.

[B38] Greenspan R. J. O’brien M. C. (1989). Genetic evidence for the role of Thy-1 in neurite outgrowth in the mouse. *J. Neurogenet.* 5 25–36. 10.3109/01677068909167262 2564888

[B39] Hachisuka A. Yamazaki T. Sawada J. Terao T. (1996). Characterization and tissue distribution of opioid-binding cell adhesion molecule (OBCAM) using monoclonal antibodies. *Neurochem. Int.* 28 373–379. 10.1016/0197-0186(95)00108-5 8740443

[B40] Harpaz Y. Chothia C. (1994). Many of the immunoglobulin superfamily domains in cell adhesion molecules and surface receptors belong to a new structural set which is close to that containing variable domains. *J. Mol. Biol.* 238 528–539. 10.1006/jmbi.1994.1312 8176743

[B41] Hashimoto T. Maekawa S. Miyata S. (2009). IgLON cell adhesion molecules regulate synaptogenesis in hippocampal neurons. *Cell Biochem. Funct.* 27 496–498. 10.1002/cbf.1600 19711485

[B42] Heinla I. Leidmaa E. Kongi K. Pennert A. Innos J. Nurk K. (2015). Gene expression patterns and environmental enrichment-induced effects in the hippocampi of mice suggest importance of Lsamp in plasticity. *Front. Neurosci.* 9:205. 10.3389/fnins.2015.00205 26136648 PMC4470440

[B43] Held W. Mariuzza R. A. (2011). Cis–trans interactions of cell surface receptors: biological roles and structural basis. *Cell. Mol. Life Sci.* 68 3469–3478. 10.1007/s00018-011-0798-z 21863376 PMC11115084

[B44] Hemperly J. J. Edelman G. M. Cunningham B. A. (1986). cDNA clones of the neural cell adhesion molecule (N-CAM) lacking a membrane-spanning region consistent with evidence for membrane attachment via a phosphatidylinositol intermediate. *Proc. Natl. Acad. Sci. U.S.A.* 83 9822–9826. 10.1073/pnas.83.24.9822 3467341 PMC387234

[B45] Hermosilla T. Muñoz D. Herrera-Molina R. Valdivia A. Muñoz N. Nham S. (2008). Direct Thy-1/αVβ3 integrin interaction mediates neuron to astrocyte communication. *Biochim. Biophys. Acta* 1783 1111–1120. 10.1016/j.bbamcr.2008.01.034 18346467 PMC2587321

[B46] Herrera-Molina R. Frischknecht R. Maldonado H. Seidenbecher C. I. Gundelfinger E. D. Hetz C. (2012). Astrocytic αVβ3 integrin inhibits neurite outgrowth and promotes retraction of neuronal processes by clustering Thy-1. *PLOS ONE* 7:e34295. 10.1371/journal.pone.0034295 22479590 PMC3316703

[B47] Horstkorte R. Schachner M. Magyar J. P. Vorherr T. Schmitz B. (1993). The fourth immunoglobulin-like domain of NCAM contains a carbohydrate recognition domain for oligomannosidic glycans implicated in association with L1 and neurite outgrowth. *J. Cell Biol.* 121 1409–1421. 10.1083/jcb.121.6.1409 8509458 PMC2119715

[B48] Hu Q. D. Ang B. T. Karsak M. Hu W. P. Cui X. Y. Duka T. (2003). F3/contactin acts as a functional ligand for notch during oligodendrocyte maturation. *Cell* 115 163–175. 10.1016/S0092-8674(03)00810-9 14567914

[B49] Innos J. Philips M. A. Leidmaa E. Heinla I. Raud S. Reemann P. (2011). Lower anxiety and a decrease in agonistic behaviour in Lsamp-deficient mice. *Behav. Brain Res.* 217 21–31. 10.1016/j.bbr.2010.09.019 20888367

[B50] Innos J. Philips M. A. Raud S. Lilleväli K. Kõks S. Vasar E. (2012). Deletion of the Lsamp gene lowers sensitivity to stressful environmental manipulations in mice. *Behav. Brain Res.* 228 74–81. 10.1016/j.bbr.2011.11.033 22155487

[B51] Itoh S. Hachisuka A. Kawasaki N. Hashii N. Teshima R. Hayakawa T. (2008). Glycosylation analysis of IgLON family proteins in rat brain by liquid chromatography and multiple-stage mass spectrometry. *Biochemistry* 47 10132–10154. 10.1021/bi8009778 18729387

[B52] Jeng C.-J. McCarroll S. A. Martin T. F. J. Floor E. Adams J. Krantz D. (1998). Thy-1 is a component common to multiple populations of synaptic vesicles. *J. Cell Biol.* 140 685–698. 10.1083/jcb.140.3.685 9456327 PMC2140167

[B53] Kasahara K. Watanabe K. Kozutsumi Y. Oohira A. Yamamoto T. Sanai Y. (2002). Association of GPI-anchored protein TAG-1 with Src-family kinase Lyn in lipid rafts of cerebellar granule cells. *Neurochem. Res.* 27 823–829. 10.1023/A:1020265225916 12374219

[B54] Kasahara K. Watanabe K. Takeuchi K. Kaneko H. Oohira A. Yamamoto T. (2000). Involvement of gangliosides in glycosylphosphatidylinositol-anchored neuronal cell adhesion molecule TAG-1 signaling in lipid rafts. *J. Biol. Chem.* 275 34701–34709. 10.1074/jbc.M003163200 10944523

[B55] Kim H. Chun Y. Che L. Kim J. Lee S. Lee S. (2017). The new obesity-associated protein, neuronal growth regulator 1 (NEGR1), is implicated in Niemann-Pick disease Type C (NPC2)-mediated cholesterol trafficking. *Biochem. Biophys. Res. Commun.* 482 1367–1374. 10.1016/j.bbrc.2016.12.043 27940359

[B56] Kiselyov V. V. Soroka V. Berezin V. Bock E. (2005). Structural biology of NCAM homophilic binding and activation of FGFR. *J. Neurochem.* 94 1169–1179. 10.1111/j.1471-4159.2005.03284.x 16045455

[B57] Koido K. Traks T. Balõtšev R. Eller T. Must A. Koks S. (2012). Associations between LSAMP gene polymorphisms and major depressive disorder and panic disorder. *Transl. Psychiatry* 2 e152. 10.1038/tp.2012.74 22892717 PMC3432189

[B58] Kuhn T. B. Stoeckli E. T. Condrau M. A. Rathjen F. G. Sonderegger P. (1991). Neurite outgrowth on immobilized axonin-1 is mediated by a heterophilic interaction with L1(G4). *J. Cell Biol.* 115 1113–1126. 10.1083/jcb.115.4.1113 1720120 PMC2289947

[B59] Kulahin N. Kristensen O. Rasmussen K. K. Olsen L. Rydberg P. Vestergaard B. (2011). Structural model and trans-interaction of the entire ectodomain of the olfactory cell adhesion molecule. *Structure* 19 203–211. 10.1016/j.str.2010.12.014 21300289

[B60] Kuroiwa K. Torikai Y. Osawa M. Nakashima T. Nakashima M. Endo H. (2012). Epitope determination of anti rat thy-1 monoclonal antibody that regulates neurite outgrowth. *Hybridoma* 31 225–232. 10.1089/hyb.2012.0002 22894774

[B61] Leifer D. Lipton S. A. Barnstable C. J. Masland R. H. (1984). Monoclonal antibody to Thy-1 enhances regeneration of processes by rat retinal ganglion cells in culture. *Science* 224 303–306. 10.1126/science.6143400 6143400

[B62] Leshchyns’ka I. Sytnyk V. (2016). Reciprocal interactions between cell adhesion molecules of the immunoglobulin superfamily and the cytoskeleton in neurons. *Front. Cell Dev. Biol.* 4:9. 10.3389/fcell.2016.00009 26909348 PMC4754453

[B63] Leshchyns’ka I. Sytnyk V. Morrow J. S. Schachner M. (2003). Neural cell adhesion molecule (NCAM) association with PKCβ 2 via βI spectrin is implicated in NCAM-mediated neurite outgrowth. *J. Cell Biol.* 161 625–639. 10.1083/jcb.200303020 12743109 PMC2172933

[B64] Leyton L. Schneider P. Labra C. V. Rüegg C. Hetz C. A. Quest A. F. G. (2001). Thy-1 binds to integrin β3 on astrocytes and triggers formation of focal contact sites. *Curr. Biol.* 11 1028–1038. 10.1016/S0960-9822(01)00262-7 11470407

[B65] Li P. Prasad S. S. Mitchell D. E. Hachisuka A. Sawada J. -Í Al-Housseini A. M. (2006). Postnatal expression profile of OBCAM implies its involvement in visual cortex development and plasticity. *Cereb. Cortex* 16 291–299. 10.1093/cercor/bhi109 15901654 PMC1351221

[B66] Liu F. Arias-Vásquez A. Sleegers K. Aulchenko Y. S. Kayser M. Sanchez-Juan P. (2007). A genomewide screen for late-onset alzheimer disease in a genetically isolated dutch population. *Am. J. Hum. Genet.* 81 17–31. 10.1086/518720 17564960 PMC1950931

[B67] Lodge A. P. Howard M. R. McNamee C. J. Moss D. J. (2000). Co-localisation, heterophilic interactions and regulated expression of IgLON family proteins in the chick nervous system. *Mol. Brain Res.* 82 84–94. 10.1016/S0169-328X(00)00184-4 11042360

[B68] Lu Z. Reddy M. V. Liu J. Kalichava A. Liu J. Zhang L. (2016). Molecular architecture of contactin-Associated protein-like 2 (CNTNAP2) and its interaction with contactin 2 (CNTN2). *J. Biol. Chem.* 291 24133–24147. 10.1074/jbc.M116.748236 27621318 PMC5104938

[B69] Lustig M. Sakurai T. Grumet M. (1999). Nr-CAM promotes neurite outgrowth from peripheral ganglia by a mechanism involving axonin-1 as a neuronal receptor. *Dev. Biol.* 209 340–351. 10.1006/dbio.1999.9250 10328925

[B70] Ma Q.-H. Futagawa T. Yang W.-L. Jiang X.-D. Zeng L. Takeda Y. (2008). A TAG1-APP signalling pathway through Fe65 negatively modulates neurogenesis. *Nat. Cell Biol.* 10 283–294. 10.1038/ncb1690 18278038

[B71] Maccarrone G. Ditzen C. Yassouridis A. Rewerts C. Uhr M. Uhlen M. (2013). Psychiatric patient stratification using biosignatures based on cerebrospinal fluid protein expression clusters. *J. Psychiatr. Res.* 47 1572–1580. 10.1016/j.jpsychires.2013.07.021 23962679

[B72] Mahanthappa N. K. Patterson P. H. (1992). Thy-1 involvement in neurite outgrowth: perturbation by antibodies, phospholipase C, and mutation. *Dev. Biol.* 150 47–59. 10.1016/0012-1606(92)90006-3 1347021

[B73] Malhotra J. D. Tsiotra P. Karagogeos D. Hortsch M. (1998). Cis-activation of L1-mediated ankyrin recruitment by TAG-1 homophilic cell adhesion. *J. Biol. Chem.* 273 33354–33359. 10.1074/jbc.273.50.33354 9837910

[B74] Manso C. Querol L. Mekaouche M. Illa I. Devaux J. J. (2016). Contactin-1 IgG4 antibodies cause paranode dismantling and conduction defects. *Brain* 139 1700–1712. 10.1093/brain/aww062 27017186

[B75] Mao X. Schwend T. Conrad G. W. (2012). Expression and localization of neural cell adhesion molecule and polysialic acid during chick corneal development. *Investig. Ophthalmol. Vis. Sci.* 53 1234–1243. 10.1167/iovs.11-8834 22281821 PMC3339905

[B76] Marg A. Sirim P. Spaltmann F. Plagge A. Kauselmann G. Buck F. (1999). Neurotractin, a novel neurite outgrowth-promoting Ig-like protein that interacts with CEPU-1 and LAMP. *J. Cell Biol.* 145 865–876. 10.1083/jcb.145.4.865 10330412 PMC2133198

[B77] Mayeux-Portas V. File S. E. Stewart C. L. Morris R. J. (2000). Mice lacking the cell adhesion molecule Thy-1 fail to use socially transmitted cues to direct their choice of food. *Curr. Biol.* 10 68–75. 10.1016/S0960-9822(99)00278-X 10662668

[B78] Mercati O. Danckaert A. Andre-Leroux G. Bellinzoni M. Gouder L. Watanabe K. (2013). Contactin 4, -5 and -6 differentially regulate neuritogenesis while they display identical PTPRG binding sites. *Biol. Open* 2 324–334. 10.1242/bio.20133343 23519440 PMC3603414

[B79] Messer A. Snodgrass G. L. Maskin P. (1984). Enhanced survival of cultured cerebellar Purkinje cells by plating on antibody to Thy-1. *Cell. Mol. Neurobiol.* 4 285–290. 10.1007/BF00733591 6395956 PMC11572875

[B80] Mikami T. Yasunaga D. Kitagawa H. (2009). Contactin-1 is a functional receptor for neuroregulatory chondroitin sulfate-E. *J. Biol. Chem.* 284 4494–4499. 10.1074/jbc.M809227200 19075012

[B81] Miyata S. Matsumoto N. Taguchi K. Akagi A. Iino T. Funatsu N. (2003a). Biochemical and ultrastructural analyses of IgLON cell adhesion molecules, Kilon and OBCAM in the rat brain. *Neuroscience* 117 645–658. 10.1016/S0306-4522(02)00873-4 12617969

[B82] Miyata S. Taguchi K. Maekawa S. (2003b). Dendrite-associated opioid-binding cell adhesion molecule localizes at neurosecretory granules in the hypothalamic magnocellular neurons. *Neuroscience* 122 169–181. 10.1016/S0306-4522(03)00609-2 14596858

[B83] Morales G. Hubert M. Brümmendorf T. Treubert U. Tárnok A. Schwarz U. (1993). Induction of axonal growth by heterophilic interactions between the cell surface recognition proteins Fll and Nr-CAM/Bravo. *Neuron* 11 1113–1122. 10.1016/0896-6273(93)90224-F 8274278

[B84] Moreland J. L. Gramada A. Buzko O. V. Zhang Q. Bourne P. E. (2005). The molecular biology toolkit (MBT): a modular platform for developing molecular visualization applications. *BMC Bioinformatics* 6:21. 10.1186/1471-2105-6-21 15694009 PMC548701

[B85] Murai K. K. Misner D. Ranscht B. (2002). Contactin supports synaptic plasticity associated with hippocampal long-term depression but not potentiation. *Curr. Biol.* 12 181–190. 10.1016/S0960-9822(02)00680-2 11839269

[B86] Must A. Tasa G. Lang A. Vasar E. Kõks S. Maron E. (2008). Association of limbic system-associated membrane protein (LSAMP) to male completed suicide. *BMC Med. Genet.* 9:34. 10.1186/1471-2350-9-34 18433483 PMC2386445

[B87] Nava C. Keren B. Mignot C. Rastetter A. Chantot-Bastaraud S. Faudet A. (2014). Prospective diagnostic analysis of copy number variants using SNP microarrays in individuals with autism spectrum disorders. *Eur. J. Hum. Genet.* 22 71–78. 10.1038/ejhg.2013.88 23632794 PMC3865413

[B88] Niethammer P. Delling M. Sytnyk V. Dityatev A. Fukami K. Schachner M. (2002). Cosignaling of NCAM via lipid rafts and the FGF receptor is required for neuritogenesis. *J. Cell Biol.* 157 521–532. 10.1083/jcb.200109059 11980923 PMC2173281

[B89] Nikolaienko R. M. Hammel M. Dubreuil V. Zalmai R. Hall D. R. Mehzabeen N. (2016). Structural basis for interactions between contactin family members and protein-tyrosine phosphatase receptor type G in neural tissues. *J. Biol. Chem.* 291 21335–21349. 10.1074/jbc.M116.742163 27539848 PMC5076805

[B90] Norenberg U. Hubert M. Brummendorf T. Tarnok A. Rathjen F. G. (1995). Characterization of functional domains of the tenascin-R (restrictin) polypeptide: cell attachment site, binding with F11, and enhancement of F11- mediated neurite outgrowth by tenascin-R. *J. Cell Biol.* 130 473–484. 10.1083/jcb.130.2.473 7615642 PMC2199939

[B91] Nosten-Bertrand M. Errington M. L. Murphy K. P. S. J. Tokugawa Y. Barboni E. Kozlova E. (1996). Normal spatial learning despite regional inhibition of LTP in mice lacking Thy-1. *Nature* 379 826–829. 10.1038/379826a0 8587606

[B92] O’Donovan M. C. Craddock N. Norton N. Williams H. Peirce T. Moskvina V. (2008). Identification of loci associated with schizophrenia by genome-wide association and follow-up. *Nat. Genet.* 40 1053–1055. 10.1038/ng.201 18677311

[B93] Ogawa J. Kaneko H. Masuda T. Nagata S. Hosoya H. Watanabe K. (1996). Novel neural adhesion molecules in the Contactin/F3 subgroup of the immunoglobulin superfamily: isolation and characterization of cDNAs from rat brain. *Neurosci. Lett.* 218 173–176. 10.1016/S0304-3940(96)13156-6 8945756

[B94] Oikawa S. Imajo S. Noguchi T. Kosaki G. Nakazato H. (1987). The carcinoembryonic antigen (CEA) contains multiple immunoglobulin-like domains. *Biochem. Biophys. Res. Commun.* 144 634–642. 10.1016/S0006-291X(87)80013-X 3579935

[B95] Oikawa S. Inuzuka C. Kuroki M. Arakawa F. Matsuoka Y. Kosaki G. (1991). A specific heterotypic cell adhesion activity between members of carcinoembryonic antigen family, W272 and NCA, is mediated by N-domains. *J. Biol. Chem.* 266 7995–8001. 2022629

[B96] Olive S. Dubois C. Schachner M. Rougon G. (2002). The F3 neuronal glycosylphosphatidylinositol-linked molecule is localized to glycolipid-enriched membrane subdomains and interacts with L1 and fyn kinase in cerebellum. *J. Neurochem.* 65 2307–2317. 10.1046/j.1471-4159.1995.65052307.x 7595520

[B97] Palmeri A. Privitera L. Giunta S. Loreto C. Puzzo D. (2013). Inhibition of phosphodiesterase-5 rescues age-related impairment of synaptic plasticity and memory. *Behav. Brain Res.* 240 11–20. 10.1016/j.bbr.2012.10.060 23174209

[B98] Pan Y. Wang K. S. Aragam N. (2011). NTM and NR3C2 polymorphisms influencing intelligence: family-based association studies. *Prog. Neuropsychopharmacol. Biol. Psychiatry* 35 154–160. 10.1016/j.pnpbp.2010.10.016 21036197

[B99] Panichareon B. Nakayama K. Thurakitwannakarn W. Iwamoto S. Sukhumsirichart W. (2012). OPCML gene as a schizophrenia susceptibility locus in Thai population. *J. Mol. Neurosci.* 46 373–377. 10.1007/s12031-011-9595-2 21833655

[B100] Paulick M. G. Bertozzi C. R. (2008). The glycosylphosphatidylinositol anchor: a complex membrane-anchoring structure for proteins. *Biochemistry* 47 6991–7000. 10.1021/bi8006324 18557633 PMC2663890

[B101] Peles E. Nativ M. Campbell P. L. Sakurai T. Martinez R. Levt S. (1995). The carbonic anhydrase domain of receptor tyrosine phosphatase β is a functional ligand for the axonal cell recognition molecule contactin. *Cell* 82 251–260. 10.1016/0092-8674(95)90312-7 7628014

[B102] Peles E. Nativ M. Lustig M. Grumet M. Schilling J. Martinez R. (1997). Identification of a novel contactin-associated transmembrane receptor with multiple domains implicated in protein-protein interactions. *EMBO J.* 16 978–988. 10.1093/emboj/16.5.978 9118959 PMC1169698

[B103] Pimenta A. F. Fischer I. Levitt P. (1996). cDNA cloning and structural analysis of the human limbic-system-associated membrane protein (LAMP). *Gene* 170 189–195. 10.1016/0378-1119(96)84698-1 8666243

[B104] Pischedda F. Piccoli G. (2015). The IgLON family member NEGR1 promotes neuronal arborization acting as soluble factor via FGFR2. *Front. Mol. Neurosci.* 8:89. 10.3389/fnmol.2015.00089 26793057 PMC4710852

[B105] Poliak S. Salomon D. Elhanany H. Sabanay H. Kiernan B. Pevny L. (2003). Juxtaparanodal clustering of Shaker-like K+ channels in myelinated axons depends on Caspr2 and TAG-1. *J. Cell Biol.* 162 1149–1160. 10.1083/jcb.200305018 12963709 PMC2172860

[B106] Poltorak M. Frye M. A. Wright R. Hemperly J. J. George M. S. Pazzaglia P. J. (1996). Increased neural cell adhesion molecule in the CSF of patients with mood disorder. *J. Neurochem.* 66 1532–1538. 10.1046/j.1471-4159.1996.66041532.x 8627309

[B107] Poot M. (2014). A candidate gene association study further corroborates involvement of contactin genes in autism. *Mol. Syndromol.* 5 229–235. 10.1159/000362891 25337070 PMC4188154

[B108] Puzzo D. Bizzoca A. Loreto C. Guida C. A. Gulisano W. Frasca G. (2015). Role of F3/contactin expression profile in synaptic plasticity and memory in aged mice. *Neurobiol. Aging* 36 1702–1715. 10.1016/j.neurobiolaging.2015.01.004 25659859

[B109] Puzzo D. Bizzoca A. Privitera L. Furnari D. Giunta S. Girolamo F. (2013). F3/Contactin promotes hippocampal neurogenesis, synaptic plasticity, and memory in adult mice. *Hippocampus* 23 1367–1382. 10.1002/hipo.22186 23939883

[B110] Qiu S. Champagne D. L. Peters M. Catania E. H. Weeber E. J. Levitt P. (2010). Loss of limbic system-associated membrane protein leads to reduced hippocampal mineralocorticoid receptor expression, impaired synaptic plasticity, and spatial memory deficit. *Biol. Psychiatry* 68 197–204. 10.1016/j.biopsych.2010.02.013 20385375 PMC2900390

[B111] Querol L. Nogales-Gadea G. Rojas-Garcia R. Martinez-Hernandez E. Diaz-Manera J. Suárez-Calvet X. (2013). Antibodies to contactin-1 in chronic inflammatory demyelinating polyneuropathy. *Ann. Neurol.* 73 370–380. 10.1002/ana.23794 23280477

[B112] Rader C. Stoeckli E. T. Ziegler U. Osterwalder T. Kunz B. Sonderegger P. (1993). Cell-cell adhesion by homophilic interaction of the neuronal recognition molecule axonin-1. *Eur. J. Biochem.* 215 133–141. 10.1111/j.1432-1033.1993.tb18015.x 8344273

[B113] Ranheim T. S. Edelman G. M. Cunningham B. A. (1996). Homophilic adhesion mediated by the neural cell adhesion molecule involves multiple immunoglobulin domains. *Proc. Natl. Acad. Sci. U.S.A.* 93 4071–4075. 10.1073/pnas.93.9.4071 8633018 PMC39488

[B114] Ranscht B. (1988). Sequence of contactin, a 130-kD glycoprotein concentrated in areas of interneuronal contact, defines a new member of the immunoglobulin supergene family in the nervous system. *J. Cell Biol.* 107 1561–1573. 10.1083/jcb.107.4.1561 3049624 PMC2115254

[B115] Reed J. McNamee C. Rackstraw S. Jenkins J. Moss D. (2004). Diglons are heterodimeric proteins composed of IgLON subunits, and Diglon-CO inhibits neurite outgrowth from cerebellar granule cells. *J. Cell Sci.* 117 3961–3973. 10.1242/jcs.01261 15265982

[B116] Rios J. C. Melendez-Vasquez C. V. Einheber S. Lustig M. Grumet M. Hemperly J. (2000). Contactin-associated protein (Caspr) and contactin form a complex that is targeted to the paranodal junctions during myelination. *J. Neurosci.* 20 8354–8364. 11069942 10.1523/JNEUROSCI.20-22-08354.2000 PMC6773165

[B117] Rubio-Marrero E. N. Vincelli G. Jeffries C. M. Shaikh T. R. Pakos I. S. Ranaivoson F. M. (2016). Structural characterization of the extracellular domain of CASPR2 and insights into its association with the novel ligand contactin1. *J. Biol. Chem.* 291 5788–5802. 10.1074/jbc.M115.705681 26721881 PMC4786715

[B118] Sabater L. Planagumà J. Dalmau J. Graus F. (2016). Cellular investigations with human antibodies associated with the anti-IgLON5 syndrome. *J. Neuroinflammation* 13 226. 10.1186/s12974-016-0689-1 27586161 PMC5007989

[B119] Sanz R. Ferraro G. B. Fournier A. E. (2015). IgLON cell adhesion molecules are shed from the cell surface of cortical neurons to promote neuronal growth. *J. Biol. Chem.* 290 4330–4342. 10.1074/jbc.M114.628438 25538237 PMC4326840

[B120] Schol-Gelok S. Janssens A. C. J. W. Tiemeier H. Liu F. Lopez-Leon S. Zorkoltseva I. V. (2010). A genome-wide screen for depression in two independent dutch populations. *Biol. Psychiatry* 68 187–196. 10.1016/j.biopsych.2010.01.033 20452571

[B121] Schroeder R. London E. Brown D. (1994). Interactions between saturated acyl chains confer detergent resistance on lipids and glycosylphosphatidylinositol (GPI)-anchored proteins: GPI-anchored proteins in liposomes and cells show similar behavior. *Proc. Natl. Acad. Sci. U.S.A.* 91 12130–12134. 10.1073/pnas.91.25.12130 7991596 PMC45390

[B122] Shimoda Y. Koseki F. Itoh M. Toyoshima M. Watanabe K. (2012). A cis-complex of NB-2/contactin-5 with amyloid precursor-like protein 1 is localized on the presynaptic membrane. *Neurosci. Lett.* 510 148–153. 10.1016/j.neulet.2012.01.026 22285261

[B123] Simon P. D. McConnell J. Zurakowski D. Vorwerk C. K. Naskar R. Grosskreutz C. L. (1999). Thy-1 is critical for normal retinal development. *Dev. Brain Res.* 117 219–223. 10.1016/S0165-3806(99)00123-6 10567740

[B124] Simons K. Ikonen E. (1997). Functional rafts in cell membranes. *Nature* 387 569–572. 10.1038/42408 9177342

[B125] Soroka V. Kolkova K. Kastrup J. S. Diederichs K. Breed J. Kiselyov V. V. (2003). Structure and interactions of NCAM Ig1-2-3 suggest a novel zipper mechanism for homophilic adhesion. *Structure* 11 1291–1301. 10.1016/j.str.2003.09.006 14527396

[B126] Stoeckli E. T. Kuhn T. B. Duc C. O. Ruegg M. A. Sonderegger P. (1991). The axonally secreted protein axonin-1 is a potent substratum for neurite growth. *J. Cell Biol.* 112 449–455. 10.1083/jcb.112.3.449 1991792 PMC2288832

[B127] Stogmann E. Reinthaler E. Eltawil S. El Etribi M. A. Hemeda M. El Nahhas N. (2013). Autosomal recessive cortical myoclonic tremor and epilepsy: association with a mutation in the potassium channel associated gene CNTN2. *Brain* 136 1155–1160. 10.1093/brain/awt068 23518707

[B128] Stohl W. Gonatas N. K. (1977). Distribution of the thy-1 antigen in cellular and subcellular fractions of adult mouse brain. *J. Immunol.* 119 422–427. 69659

[B129] Struyk A. F. Canoll P. D. Wolfgang M. J. Rosen C. L. D’Eustachio P. Salzer J. L. (1995). Cloning of neurotrimin defines a new subfamily of differentially expressed neural cell adhesion molecules. *J. Neurosci.* 15 2141–2156. 7891157 10.1523/JNEUROSCI.15-03-02141.1995 PMC6578143

[B130] Südhof T. C. (2008). Neuroligins and neurexins link synaptic function to cognitive disease. *Nature* 455 903–911. 10.1038/nature07456 18923512 PMC2673233

[B131] Sytnyk V. Leshchyns’ka I. Schachner M. (2017). Neural cell adhesion molecules of the immunoglobulin superfamily regulate synapse formation, maintenance, and function. *Trends Neurosci.* 40 295–308. 10.1016/j.tins.2017.03.003 28359630

[B132] Taheri M. Saragovi U. Fuks A. Makkerh J. Mort J. Stanners C. P. (2000). Self recognition in the Ig superfamily: identification of precise subdomains in carcinoembryonic antigen required for intercellular adhesion. *J. Biol. Chem.* 275 26935–26943. 10.1074/jbc.M909242199 10864933

[B133] Takamori S. Holt M. Stenius K. Lemke E. A. Grønborg M. Riedel D. (2006). Molecular anatomy of a trafficking organelle. *Cell* 127 831–846. 10.1016/j.cell.2006.10.030 17110340

[B134] Tamási V. Petschner P. Adori C. Kirilly E. Ando R. D. Tothfalusi L. (2014). Transcriptional evidence for the role of chronic venlafaxine treatment in neurotrophic signaling and neuroplasticity including also glutatmatergic- and insulin-mediated neuronal processes. *PLOS ONE* 9:e113662. 10.1371/journal.pone.0113662 25423262 PMC4244101

[B135] Tassano E. Gamucci A. Celle M. E. Ronchetto P. Cuoco C. Gimelli G. (2015). Clinical and molecular cytogenetic characterization of a de novo interstitial 1p31.1p31.3 deletion in a boy with moderate intellectual disability and severe language impairment. *Cytogenet. Genome Res.* 146 39–43. 10.1159/000431391 26112959

[B136] Traka M. Goutebroze L. Denisenko N. Bessa M. Nifli A. Havaki S. (2003). Association of TAG-1 with Caspr2 is essential for the molecular organization of juxtaparanodal regions of myelinated fibers. *J. Cell Biol.* 162 1161–1172. 10.1083/jcb.200305078 12975355 PMC2172849

[B137] Treubert U. Brümmendorf T. (1998). Functional cooperation of beta1-integrins and members of the Ig superfamily in neurite outgrowth induction. *J. Neurosci.* 18 1795–1805. 9465004 10.1523/JNEUROSCI.18-05-01795.1998 PMC6792609

[B138] Van Daalen E. Kemner C. Verbeek N. E. Van Der Zwaag B. Dijkhuizen T. Rump P. (2011). Social responsiveness scale-aided analysis of the clinical impact of copy number variations in autism. *Neurogenetics* 12 315–323. 10.1007/s10048-011-0297-2 21837366 PMC3215885

[B139] Varma R. Mayor S. (1998). GPI-anchored proteins are organized in submicron domains at the cell surface. *Nature* 394 798–801. 10.1038/29563 9723621

[B140] Veerappa A. M. Saldanha M. Padakannaya P. Ramachandra N. B. (2013). Genome-wide copy number scan identifies disruption of PCDH11X in developmental dyslexia. *Am. J. Med. Genet. Part B Neuropsychiatr. Genet.* 162 889–897. 10.1002/ajmg.b.32199 24591081

[B141] Walmod P. S. Kolkova K. Berezin V. Bock E. (2004). Zippers make signals: NCAM-mediated molecular interactions and signal transduction. *Neurochem. Res.* 29 2015–2035. 10.1007/s11064-004-6875-z 15662836

[B142] Wang W. Karagogeos D. Kilpatrick D. L. (2011). The effects of Tag-1 on the maturation of mouse cerebellar granule neurons. *Cell. Mol. Neurobiol.* 31 351–356. 10.1007/s10571-010-9641-6 21191645 PMC3117589

[B143] Williams A. F. Barclay A. N. (1988). The immunoglobulin superfamily—domains for cell surface recognition. *Annu. Rev. Immunol.* 6 381–405. 10.1146/annurev.iy.06.040188.002121 3289571

[B144] Williams A. F. Gagnon J. (1982). Neuronal cell Thy-1 glycoprotein: homology with immunoglobulin. *Science* 216 696–703. 10.1126/science.6177036 6177036

[B145] Yamada M. Hashimoto T. Hayashi N. Higuchi M. Murakami A. Nakashima T. (2007). Synaptic adhesion molecule OBCAM; synaptogenesis and dynamic internalization. *Brain Res.* 1165 5–14. 10.1016/j.brainres.2007.04.062 17658490

[B146] Yamagata M. Sanes J. R. (2012). Expanding the immunoglobulin superfamily code for laminar specificity in retina: expression and role of contactins. *J. Neurosci.* 32 14402–14414. 10.1523/JNEUROSCI.3193-12.2012 23055510 PMC3488879

[B147] Ye H. Tan Y. L. J. Ponniah S. Takeda Y. Wang S.-Q. Schachner M. (2008). Neural recognition molecules CHL1 and NB-3 regulate apical dendrite orientation in the neocortex via PTP alpha. *EMBO J.* 27 188–200. 10.1038/sj.emboj.7601939 18046458 PMC2206121

[B148] Yoshihara Y. Kawasaki M. Tamada A. Nagata S. Kagamiyama H. Mori K. (1995). Overlapping and differential expression of BIG-2, BIG-1, TAG-1, and F3: four members of an axon-associated cell adhesion molecule subgroup of the immunoglobulin superfamily. *J. Neurobiol.* 28 51–69. 10.1002/neu.480280106 8586965

[B149] Yoshihara Y. Kawasaki M. Tani A. Tamada A. Nagata S. Kagamiyama H. (1994). BIG-1: a new TAG-1/F3-related member of the immunoglobulin superfamily with neurite outgrowth-promoting activity. *Neuron* 13 415–426. 10.1016/0896-6273(94)90357-3 8060619

[B150] Yoshihara Y. Oka S. Ikeda J. Mori K. (1991). Immunoglobulin superfamily molecules in the nervous system. *Neurosci. Res.* 10 83–105. 10.1016/0168-0102(91)90033-U 1710044

[B151] Zacco A. Cooper V. Chantler P. D. Fisher-Hyland S. Horton H. L. Levitt P. (1990). Isolation, biochemical characterization and ultrastructural analysis of the limbic system-associated membrane protein (LAMP), a protein expressed by neurons comprising functional neural circuits. *J. Neurosci.* 10 73–90. 1688937 10.1523/JNEUROSCI.10-01-00073.1990 PMC6570356

[B152] Zacharias U. (2002). Tenascin-R induces actin-rich microprocesses and branches along neurite shafts. *Mol. Cell. Neurosci.* 21 626–633. 10.1006/mcne.2002.1203 12504595

[B153] Zhou H. Fuks A. Alcaraz G. Bolling T. J. Stanners C. P. (1993). Homophilic adhesion between Ig superfamily carcinoembryonic antigen molecules involves double reciprocal bonds. *J. Cell Biol.* 122 951–960. 10.1083/jcb.122.4.951 8349740 PMC2119577

[B154] Zhukareva V. Levitt P. (1995). The limbic system-associated membrane protein (LAMP) selectively mediates interactions with specific central neuron populations. *Development* 121 1161–1172. 7743928 10.1242/dev.121.4.1161

[B155] Zuellig R. A. Rader C. Schroeder A. Kalousek M. B. Von Bohlen und Halbach F. Osterwalder T. (1992). The axonally secreted cell adhesion molecule, axonin-1. Primary structure, immunoglobulin-like and fibronectin-type-III-like domains and glycosyl-phosphatidylinositol anchorage. *Eur. J. Biochem.* 204 453–463. 10.1111/j.1432-1033.1992.tb16655.x 1311675

